# Phytochemical investigations and antioxidant potential of roots of *Leea macrophylla* (Roxb.)

**DOI:** 10.1186/s13104-017-2503-2

**Published:** 2017-07-06

**Authors:** Zobaer Al Mahmud, Sitesh C. Bachar, Choudhury Mahmood Hasan, Talha Bin Emran, Nazmul Qais, Mir Muhammad Nasir Uddin

**Affiliations:** 1 Department of Clinical Pharmacy and Pharmacology, Faculty of Pharmacy, University of Dhaka, Dhaka, 1000 Bangladesh; 2 Department of Pharmaceutical Technology, Faculty of Pharmacy, University of Dhaka, Dhaka, 1000 Bangladesh; 3 Department of Pharmaceutical Chemistry, Faculty of Pharmacy, University of Dhaka, Dhaka, 1000 Bangladesh; 4 Laboratory of Vaccinology and Applied Immunology, Kanazawa University School of Pharmacy, Kakuma-machi, Kanazawa, 920-1192 Japan; 5 Department of Pharmacy, BGC Trust University Bangladesh, Chittagong, 4000 Bangladesh; 6 Department of Biochemistry and Molecular Biology, University of Chittagong, Chittagong, 4331 Bangladesh; 7 Department of Pharmacy, University of Chittagong, Chittagong, 4331 Bangladesh

**Keywords:** *Leea macrophylla*, Spectroscopic analysis, Antioxidant potential, Phytochemical, Compound isolation

## Abstract

**Background:**

Oleanolic acid (NZ-15), 7 α, 28-olean diol (NZ-38) and Stigmasterol (NZ-14) were isolated from the ethanolic extracts of the roots of *Leea macrophylla* (Family: Leeaceae) by using chromatographic analysis. This is the first report of isolation of these compounds from this plant. Their structures were constructed by spectroscopic analysis and by comparing the data with the published one. Subsequently the ethanolic extract was fractionated with two organic solvents and all the fractions were studied to evaluate their in vitro antioxidant property.

**Methods:**

The ethanolic extract was fractionated with two organic solvents and all the fractions were studied to evaluate their in vitro antioxidant property by DPPH free radical scavenging assay, superoxide anion radical scavenging assay, nitric oxide radical scavenging assay, and reducing power assay.

**Results:**

In the DPPH free radical scavenging assay and superoxide radical scavenging assay, the ethyl acetate soluble fraction of ethanolic extract revealed the highest free radical scavenging activity with IC_50_ value of 2.65 and 155.62 μg/ml, respectively as compared to standard ascorbic acid (IC_50_ value of 5.8 and 99.66 μg/ml). Ethyl acetate fraction also possessed highest reducing power activity with an EC50 value of 15.27 μg/ml compared to ascorbic acid (EC_50_ 0.91 μg/ml). On the other hand, the carbon tetrachloride fraction exhibited most significant NO scavenging activity with IC_50_ value of 277.8 μg/ml that was even higher than that of standard ascorbic acid (IC_50_ value 356.04 μg/ml). In addition, the total phenolic contents of these extract and fractions were evaluated using Folin-Ciocalteu reagent and varied from 7.93 to 50.21 mg/g dry weight expressed as gallic acid equivalents (GAE).

**Conclusions:**

This study showed that different extracts of roots of *L. macrophylla* possess potential DPPH, superoxide, and NO free radical scavenging activities. The antioxidant activities of the plant extracts might be due to the presence of oleanolic acid, oleanolic acid derivative 7 α, 28-olean diol and stigmasterol.

## Background

One of the most important mechanisms affected in several diseases such as cancer, diabetes, aging and neurodegenerative disorders is the antioxidant mechanism that is developed for the protection of living systems against oxidative injuries caused by reactive oxygen and nitrogen species [1]. Reactive oxygen species (ROS) are various forms of activated oxygen, which include free radicals such as superoxide ions (${\text{O}}_{2}^{ - }$), and reactive hydroxyl radicals (OH^−^), as well as non-free radical species such as hydrogen peroxide (H_2_O_2_) [2, 3]. In living organisms, various ROS can form in different ways. Normal aerobic respiration stimulates polymorphonuclear leukocytes and macrophages, and peroxisomes appear to be the main endogenous sources of most of the oxidants produced by cells [4]. When produced in excess, ROSs can cause tissue injury. In addition, oxidative stress is now thought to make a significant contribution to all inflammatory diseases (arthritis, vasculitis, glomerulonephritis, lupus erythematous, adult respiratory diseases syndrome), ischemic diseases (heart diseases, stroke, intestinal ischema), hemochromatosis, acquired immunodeficiency syndrome, emphysema, organ transplantation, gastric ulcers, hypertension and preeclampsia, neurological disorder (Alzheimer’s disease, Parkinson’s disease, muscular dystrophy), alcoholism, smoking-related diseases, and many others [5, 6]. Although our body has enzymatic and nonenzymatic endogenous antioxidant systems, they are insufficient to protect against free radical damage. Therefore, exogenous antioxidants play an important role in maintaining human health status [1]. There are some synthetic antioxidant compounds, such as butylated hydroxytoluene (BHT) and butylated hydroxyanisole (BHA), commonly used in processed foods. However it has been suggested that these compounds have some side effects [7–9]. Therefore, the need to find natural and safe sources of antioxidants has notably increased. The medicinal properties of plants have been investigated in recent scientific developments through the world, due to their potent antioxidant activities, no side effects and economic viability. Among natural antioxidants, phenolic antioxidants are in the forefront since all the phenolic classes (simple phenolics, phenolic acids, anthocyanins, hydroxycinnamic acid derivatives, and flavonoids) have the structural requirements of free radical scavengers and antioxidants. So current research is now directed towards finding naturally occurring antioxidant of herbal drugs.

*Leea macrophylla* Roxburgh (Family: Leeaceae; Bengali name: Dholsamudra, Dinda, Hatikana) is a robust herb or shrub, is widely distributed in Dinajpur, Savar and Chittagong hill tracts of Bangladesh, Yunnan of China, Cambodia, India, Laos, Myanmar, Nepal and Thailand. The roots of the plant have been used traditionally in fracture and healing cut injury and to allay pain. It has astringent, styptic and antiseptic activity [10]. Though, the plant has a lot of ethnopharmacological use, there have been no scientific study on the chemical and pharmacological properties of roots of the plant. Previously, we have reported antinociceptive activities of different extracts of roots of *L. macrophylla* in swiss albino [11]. There are no scientific reports about antioxidant potentials of roots of *L. macrophylla*. Therefore, the purpose of this study was to evaluate different extractives of roots of *L. macrophylla* as new potential sources of natural antioxidants and phenolic compounds. Antioxidant activities of different extract of the plant were also evaluated by using DPPH free radical scavenging assay, superoxide anion radical scavenging assay, nitric oxide radical scavenging assay, and reducing power assay.

## Methods

### Chemicals and reagents

1,1-Diphenyl-2-picrylhydrazyl (DPPH), L-Ascorbic acid, Gallic acid, Quercetin, Folin-Ciocalteu reagent, Nitroblue tetrazolium, Sodium carbonate, EDTA, Trichloroacetic acid, Potassium iodide (KI), Sodium acetate, Hydroxylamine hydrochloride, and Tween-80 were purchased from Sigma Chemical Co. (St. Louis, MO, USA). All other reagents and chemicals were of BDH and E Merck analytical grade.

### General experimental procedures

For vacuum liquid chromatography (VLC) and gel permeation chromatography columns were packed by kieselgel 60H and Sephadex (LH-20), respectively. Analytical thin layer chromatography (TLC) was performed on precoated (TLC Silica gel 60 F254-Merxk KGaA) plates using UV light, vanillin/H_2_SO_4_ reagents to visualize the spots. RV10 Basic (IKA, Germany) was used for rotary evaporation. Nuclear magnetic resonance (NMR) spectra in duterated chloroform (CDCL_3_) were recorded on a Bruker Avance 400 MHz NMR spectrometer.

### Preparation of plant extracts

The roots of *L. macrophylla were* collected from Dinajpur, Bangladesh in September, 2009. One voucher specimen has been deposited in Bangladesh national Herbarium (DACB Accession No. 35293). The dried and powdered roots (1000 g) of the plant were extracted by maceration using 4 L of ethanol. The concentrated ethanol extract was successively partitioned by pet ether, carbon tetrachloride and ethyl acetate.

### Isolation and identification of compounds

15 g of the dried ethanolic extract of roots of *L. macrophylla* was subjected to vacuum liquid chromatography (VLC) for fractionation over silica gel (kieselgel 60H, 70–230 mesh) with a mobile phase of n-hexane: ethyl acetate (100 ml of each) of 100:0, 90:10, 80:20, 70:30, 60:40, 50:50, 40:60, 30:70, 20:80, 10:90 and 0:100 (v/v). A total of 24 fractions were collected and all the VLC fractions were screened by TLC under UV light and by spraying with vanillin-sulphuric acid reagent. Depending on the TLC behaviour fractions (9–12) and (13–18) were subjected to gel permeation chromatography (packed with Sephadex LH-20) for further fractionation. The column was then eluted with the solvent systems as Table 1 and a total of 50 column fractions were collected in test tubes each containing 1 ml approximately.

**Table 1:** Solvent systems used in Sephadex column analysis of VLC fraction

Fraction No.	Solvent system	Proportion	Volume collected (ml)
1–24	Hexane:dichloromethane:methanol	2:5:1	80
25–33	Methanol:dichloromethane	9:1	30
34–40	Methanol:dichloromethane	1:1	20
41–50	Methanol	100%	50

All the column fractions were screened by TLC under UV light and by spraying with vanillin-sulphuric acid reagent. Depending on the TLC behaviour sub-fractions (21–25) of VLC fraction (9–12) and sub-fractions (22–24) of VLC fraction (13–18) were taken for further investigation. Compound NZ-14 and NZ-15 were isolated and purified from the column fractions (21–25) of VLC fraction (9–12) and compound NZ-38 was isolated from the column fractions (22–24) of VLC fraction (13–18). All the compounds were characterized by spectroscopic analysis. By comparing the ^1^H NMR data of the compounds with reported data the compounds were identified.

### Determination of the total phenolic content

Total phenolic content of the plant extracts *was* determined spectrophotometrically employing the method as described by Wolfe et al. [12] and Yu et al. [13] involving Folin–Ciocalteu reagent as oxidizing agent and gallic acid as standard. To An aliquot of the extract (0.5 ml) solution (conc. 1 mg/ml), 2.5 ml of Folin–Ciocalteu reagent (diluted 10 times with water) and 2.0 ml of Na_2_CO_3_ (7.5% w/v) solution was added. The mixture was incubated for 20 min at room temperature for color development. After 20 min the absorbance was measured at 760 nm by UV-spectrophotometer and using the standard curve prepared from gallic acid solution with different concentration, the total phenolic content of the sample was measured. The phenolic contents of the sample were expressed as mg of GAE (gallic acid equivalent)/g of the extract from the calibration curve using the equation:

$$ {\text{y}} = 0.0162{\text{x}} + 0.0215,\quad {\text{R}}^{2} = 0.9985 $$

where x was the absorbance and y was the Gallic acid equivalent (mg/g).

### DPPH free radical scavenging activity

The free radical scavenging activities of the plant extracts on the stable radical 1,1-diphenyl-2-picrylhydrazyl (DPPH) was estimated by the method described by Feresin et al. [14]. 2.0 ml of a methanol solution of the sample (extractives/control) at different concentration (500–0.977 μg/ml) were mixed with 3.0 ml of a DPPH methanol solution (20 μg/ml). After 30 min reaction period at room temperature in dark place the absorbance was measured at 517 nm against methanol as blank by UV spectrophotometer.

Inhibition of free radical DPPH in percent (I %) was calculated as follows:

$$ {\text{I}}\,\% = (1 - {\text{A}}_{\text{sample}} /{\text{A}}_{\text{blank}} ) \times 100 $$

where A_blank_ is the absorbance of the control reaction (containing all reagents except the test material). Extract concentration providing 50% inhibition (IC_50_) was calculated from the graph plotted inhibition percentage against extract concentration.

### Superoxide radical scavenging activity

Assay for the super oxide radical scavenging activity was based on the capacity of the sample to inhibit blue formazan formation by scavenging the superoxide radicals generated in Hydroxylamine-sodium carbonate-EDTA-NBT system [15]. 1 ml of 50 mM sodium carbonate, 0.4 ml of 24 µM NBT and 0.2 ml of 0.1 mM EDTA were taken in a test tube. Then 100 µl of plant extract (conc. ranging from 1000 to 7.513 µg/ml) was added to the test tube. Reaction was initiated by adding 0.4 ml of 1 mM hydroxylamine hydrochloride. The reaction mixture was incubated for 5 min at ambient temperature. After incubation, the absorbance at 562 nm was measured against an appropriate blank to determine the quantity of formazan generated.

The percentage inhibition of superoxide anion generation was calculated by the following formula:

$$ {\text{I}}\,\% = (1 - {\text{A}}_{\text{sample}} /{\text{A}}_{\text{blank}} ) \times 100 $$

where A_blank_ is the absorbance of the control reaction (containing all reagents except the test material). The decrease in absorbance at 562 nm with the plant extracts and the reference compound indicates their abilities to quench super oxide radicle- in the reaction mixture. Extract concentration providing 50% inhibition (IC_50_) was calculated from the graph plotted inhibition percentage against extract concentration.

### Nitric oxide (NO) scavenging assay

Nitric oxide (NO) scavenging activity was measured spectrophotometrically [16]. Sodium nitroprusside in aqueous solution at physiological pH spontaneously generate nitric oxide, which interacts with oxygen to produce nitrite ions determined by the use of Griess reagents. A volume of 2 ml of 10 mM sodium nitroprusside and 0.5 ml of phosphate buffer saline (pH 7.4) were mixed with 0.5 ml of plant extract and ascorbic acid individually at various concentrations (500–3.9 µg/ml). The reaction mixture was incubated at 25 °C for 150 min. After 150 min, 0.5 ml of incubation solution was withdrawn and mixed with 1 ml of sulfanilic acid reagent (0.33 in 20% glacial acetic acid) and allowed to stand for 5 min at room temperature for completing diazotization. Then 1 ml of 0.1% w/v napthylethylenediamine dihydrochloride was added, mixed well and the mixture was incubated at room temperature for 30 min. The absorbance was taken at 540 nm. The amount of nitric oxide (NO∙) radical inhibited by the extract was calculated using the following equation:

$$ {\text{I}}\,\% = (1 - {\text{A}}_{\text{sample}} /{\text{A}}_{\text{blank}} ) \times 100 $$

where A_blank_ is the absorbance of the control reaction (containing all reagents except the test material).

### Reducing power assay

The reducing power of the ethanol extract was determined by the method of Dehpour et al. [17]. A volume of 1.0 ml of the extract prepared in methanol (concentration 250–7.8125 µg/ml) and ascorbic acid were mixed individually to the mixture containing 2.5 ml of 0.2 M phosphate buffer (pH 6.6) and 2.5 ml of potassium ferricyanide [K_3_Fe(CN)_6_] (1% w/v). The resulting mixture was incubated at 50 °C for 20 min, followed by the addition of 2.5 ml of trichloroacetic acid (1% w/v). The samples were then centrifuged at 4000 rpm for 15 min. The upper layer of the solution (2.5 ml) was mixed with 2.5 ml of distilled water and 0.5 ml of ferric chloride (0.5% w/v). The absorbance was measured at 700 nm against a blank sample. Incubation with water in place of additives was used as blank while l-ascorbic acid was used as positive control. Increased absorbance of the reaction mixture indicated higher reducing power of the plant extract. The reducing power (%) was calculated based on the following formula:

$$ {\text{Reducing power}}\;(\% ) = [({\text{A}}_{\text{T}} - {\text{A}}_{\text{B}} )/{\text{A}}_{\text{T}} ] \times 100 $$

where A_B_ and A_T_ are the absorbance of blank and plant material respectively. Effective concentration, EC_50_ value of each extract was estimated from the plotted graph of percentage reducing power versus concentration of extract.

## Results and discussion

### Characterization of compounds

**Fig. 1:**
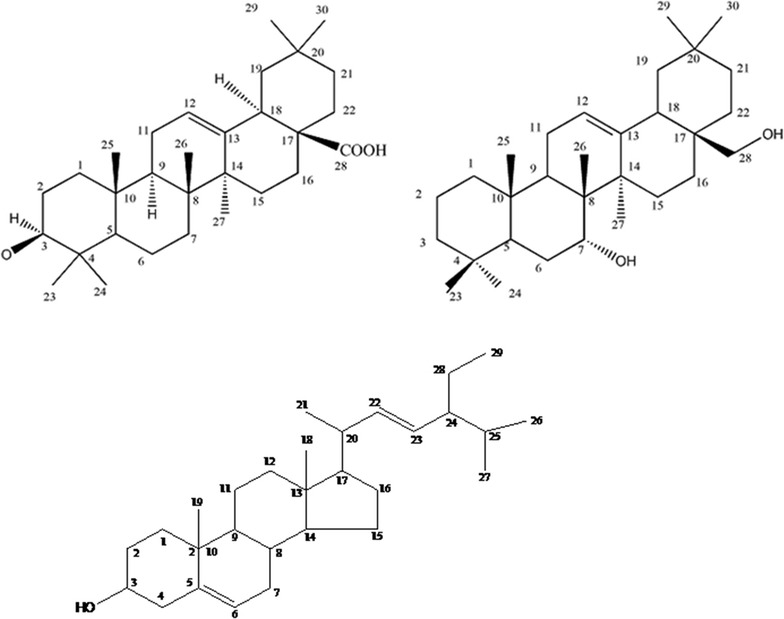
Chemical structures of compounds isolated from roots of *L. macrophylla*

Repeated chromatographic separation and purification of the different VLC fraction of crude ethanolic extract of roots of *L. macrophylla* afforded two triterpenoid, oleanolic acid, 7-α, 28-olean and a sterol, stigmasterol. The structures of the compounds were determined by spectroscopic studies (Fig. 1).

### Characterization of NZ-15 as oleanolic acid (3β-hydroxyolean-12-en-28-oic acid)

NZ-15 was obtained as white amorphous powder. It appeared as violet color on TLC after spraying the developed plate with vanillin-sulphuric acid followed by heating at 110 °C for several minutes. Comparison of ^1^H NMR spectrum of NZ-15 with those existing in the literature indicated that the compound is the pentacyclic triterpenoid oleanolic acid (3β)-3-hydroxyolean-12-en-28-oic acid) [18]. The ^1^H NMR spectrum (500 MHz, CDCl_3_, TMS) of NZ-15 showed the presence of seven methyl singlets at δ 0.75, 0.77, 0.89,0.90, 0.92, 0.97 and 1.13 on an oleanane skeleton i.e. the compound must be a pentacyclic triterpenoid. A characteristics double doublet of one proton at δ 2.81 (J = 13.6, 4.0 Hz) and a triplet of one olefinic proton at δ 5.28 (J = 3.2 Hz) were assigned to H-18 and H-12 respectively, suggesting an olean-12-ene skeleton. One methine proton at δ 3.21(1H, dd, J = 11.2 and 4.4 Hz) showed that the compound NZ-15 has at least one hydroxyl group. The coupling constant of this methine proton indicates that the hydroxyl function must be in axial position. The above spectral features were similar to the ones reported for oleanolic acid represented in Table 2.

**Table 2:** Comparison of ^1^H NMR spectral data of NZ-15 and Oleanolic acid [18]

Proton	δ_H_ in ppm	
	NZ-15 (CDCl_3_, TMS, 500 MHz)	Oleanolic acid (CDCl_3_, TMS, 500 MHz)
H-3	3.21 (^1^H, dd, J = 11.2, 4.4 Hz)	3.23 (^1^H, dd, J = 11.2, 4.4 Hz)
H-12	5.28 (1H, t, J = 3.2 Hz)	5.27 (^1^H, t, J = 3.5 Hz)
H-18	2.81 (^1^H, dd, J = 13.6, 4.0 Hz)	2.82 (^1^H, dd, J = 13.2, 3.6 Hz)
H-23	0.98 (3H, s)	0.98 (3H, s)
H-24	0.77 (3H, s)	0.77 (3H, s)
H-25	0.89 (3H, s)	0.90 (3H, s)
H-26	0.75 (3H, s)	0.75 (3H, s)
H-27	1.13 (3H, s)	1.13 (3H, s)
H-29	0.90 (3H, s)	0.91 (3H, s)
H-30	0.92 (3H, s)	0.93 (3H, s)

On this basis, the identity of NZ-15 was confirmed as oleanolic acid. Although oleanolic acid has been reported from various other plant species, this is the first report of its occurrence from *L. macrophylla.*

### Characterization of NZ-38 as 7 α, 28-olean diol

NZ-38 was obtained as white crystal from the roots of *L. macrophylla.* It gave pink color on the TLC plate after spraying with vanillin-sulphuric acid followed by heating at 110 °C for several minutes. The ^1^H NMR (CDCl_3_, 400 MHz, TMS) spectrum of compound NZ-38 showed seven three protons singlet at δ 0.77, δ 0.88 (2XCH_3_, s), δ 0.90, δ 0.92, δ 0.95 and at δ 1.13 which indicates that the compound must be a pentacyclic triterpenoid in nature having a oleanane skeleton. A double doublet of one proton at δ 2.82 (^1^H, dd, J = 10.0 and 2.0 Hz), one methine proton at δ 3.63 (^1^H, dd, J = 8.0 and 7.6 Hz) and one olefinic proton at δ 5.29 (^1^H, bt) were observed in which δ 2.82 and δ 5.29 were assigned to Hα-18 and H-12 respectively, suggesting an olean-12-ene skeleton. Comparison of ^1^H NMR spectrum of NZ-38 with that of 3β-hydroxyolean-12-en-28-oic acid indicated that NZ-38 should be an olean diol that are shown in Table 3 [18].

**Table 3:** Comparison of ^1^H NMR spectral data of NZ-38 and Oleanolic acid [18]

Proton	δ_H_ in ppm	
	NZ-15 (CDCl_3_, TMS, 500 MHz)	Oleanolic acid (CDCl_3_, TMS, 500 MHz)
H-3	3.63 (^1^H, dd,J = 8.0 & 7.6 Hz)^a^	3.23 (^1^H, dd, J = 11.2,4.4 Hz)
H-12	5.28 (^1^H, t, J = 3.2 Hz)	5.27 (^1^H, t, J = 3.5 Hz)
H-18	2.81 (^1^H, dd, J = 13.6,4.0 Hz)	2.82 (^1^H, dd, J = 13.2, 3.6 Hz)
H-23	0.98 (3H, s)	0.98 (3H, s)
H-24	0.77 (3H, s)	0.77 (3H, s)
H-25	0.89 (3H, s)	0.90 (3H, s)
H-26	0.75 (3H, s)	0.75 (3H, s)
H-27	1.1 3 (3H, s)	1.13 (3H, s)
Ha-28	3.72 (^1^H, d, J = 10.4 Hz)	
Hb-28	3.42 (^1^H, d, J = 10.0 Hz)	
H-29	0.90 (3H, s)	0.91 (3H, s)
H-30	0.92 (3H, s)	0.93 (3H, s)

^a^The position of this proton must he sifted elsewhere in the olean skeleton

The methine proton at δ 3.63 (^1^H, dd, J = 8.0 and 7.6 Hz) showed that the 3-OH group which is usually located at C-3 on olean skeleton [the corresponding chemical shift for H-3 is δ 3.21 (dd, J = 11.2 and 4.4 Hz), [18] should be shifted from the usual location. The spectral pattern indicated that the oxymethine proton is equatorial and coupled with two adjacent protons. Hence the location of the hydroxyl function can be restricted to C-1, C-3, C-7, C-15, C-16, C-21 or C-22. The possibility of C-3 was ruled out due to the large deviance of the chemical shift value from the usual δ value of 3.21. Usually the chemical shift value (δ_H_) for Me-26 and Me-29 on oleanane skeleton are δ 0.75 and δ 0.87 [18] but in case of ^1^H NMR spectrum of NZ-38 the two methyl protons of Me-26 and Me-29 merged into the δ_H_ value of δ 0.88. So there is only one significant shift of a methyl group value from the literature value was observed- the chemical shift value for Me-26 shifted to 0.88 from the literature value of 0.75. So the most possible location of the hydroxyl function can be placed at C-7α position which justifies the more deshielded δ value of the oxymethine proton. As the coupling constant of methine proton is 8 and 7.6 Hz it must be equatorial to the coupled hydrogen or in β orientation. The chemical shift value δ 3.42 (^1^H, d, J = 10.0) and δ 3.72 (^1^H, d, J = 10.4 Hz) were assigned to two protons H_a_-28 and H_b_-28 attached to the OH function of C-28. Considering all these spectral features on the basis of above discussion the structure of the compound NZ-38 was identified as 7 α, 28-olean diol.

### Characterization of NZ-14 as stigmasterol

#### Stigmasterol (NZ-14)

White needles; ^1^H NMR (400 MHz, CDCl3): δ 5.37 (^1^H, m, H-6), 5.15 (^1^H, dd, J = 14.4, 8.4 Hz, H- 22), 5.03 (^1^H, dd, J = 14.4, 8.4 Hz, H- 23), 3.52 (^1^H, m, H-3), 1.03 (3H, s, CH3-10), 0.94 (3H, d, J = 6.4 Hz, CH3-20), 0.85 (3H, d, J = 7.4 Hz, CH3-27), 0.83 (3H, d, J = 7.4 Hz, CH3-26), 0.67 (3H, s, CH3-13).

NZ-14 was obtained as white needle shaped crystal from the ethanolic extract of roots of *L. macrophylla*. It was characterized as stigmasterol by comparing the ^1^H NMR data mentioned above with those published for the compound [19].

**Table 4:** Total phenolic content of *Leea macrophylla*

Sample	Conc (µg/ml)	Absorbance			Gallic acid equivalent (µg/mg)
		A1	A2	Mean	
EELMR	250	0.243	0.257	0.249	14.04
CCFLMR	250	0.153	0.147	0.15	7.93
EAFLMR	250	0.801	0.87	0.835	50.21

### Total phenolic content

The amount of total phenolic content differs in different extractives of *L. macrophylla* and ranged from 7.93 mg of GAE/gm of extractives to 50.21 mg of GAE/gm of extractives of *L. macrophylla* (Table 4). Among all extractives the highest phenolic content was found in EALMR (50.21 mg of GAE/g of extractives). The phenolic compounds exert their antioxidant properties by redox reaction, which can play an important role in absorbing and neutralizing free radicals, quenching singlet and triplet oxygen, or decomposing peroxides.

### DPPH radical scavenging activity

**Fig. 2:**
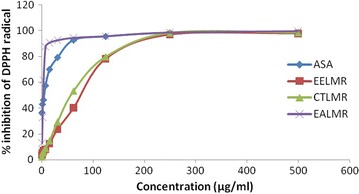
DPPH free radical scavenging activity of different extracts of roots of *L. macrophylla.* EELMR: The values are the average of duplicate experiments. Ethanolic extract of *L. macrophylla* roots; *CTLMR* carbon tetrachloride soluble partitionate, *EALMR* ethyl acetate soluble partitionate, *ASA* ascorbic acid

In this investigation, ethyl acetate fraction of roots of *L. macrophylla* (EALMR) showed the highest DPPH radical scavenging activity with IC_50_ value 2.65 μg/ml which is even higher than that of the ascorbic acid (5.8 μg/ml) that is shown in Table 5 and represent in Fig. 2.

**Table 5:** IC_50_ values and EC_50_ (μg/ml) of the standard and partitionates of roots of *L. macrophylla*

Sample	IC_50_ (μg/ml)			EC_50_ (μg/ml)
	DPPH radical assay	Superoxide radical scavenging assay	NO radical scavenging assay	Reducing power assay
EELMR	46.41	1281.7	210.12	88.1
CTLMR	39.75	222.5	277.8	58.03
EALMR	2.65	155.62	407.70	15.27
ASA	5.8	99.66	356.06	0.91

Crude ethanolic extract (IC_50_ 46.41) and CCl_4_ fraction (IC_50_ 39.75) of the roots of *L. macrophylla* also exhibited strong antioxidant potential. The results obtained from this investigation showed positive correlation between the total phenolic contents and antioxidant potential of the extractives. For example highest amount of phenolic content was found in ethyl acetate fraction of roots of *L. macrophylla* (50.21 mg of GAE/g of extracts) and this fraction showed most potent antioxidant activity (IC_50_ = 2.65 μg/ml). Phytochemical components, especially phenolic compounds such as flavonoids, phyenyl propanoids, phenolic acids, tannins, etc., are very important components for the free radical scavenging and antioxidant activities of plants. Polyphenols are generally of the chemical patterns; phenolic groups react as hydrogen donors and neutralize the free radicals [20].

### Superoxide radical scavenging activity

**Fig. 3:**
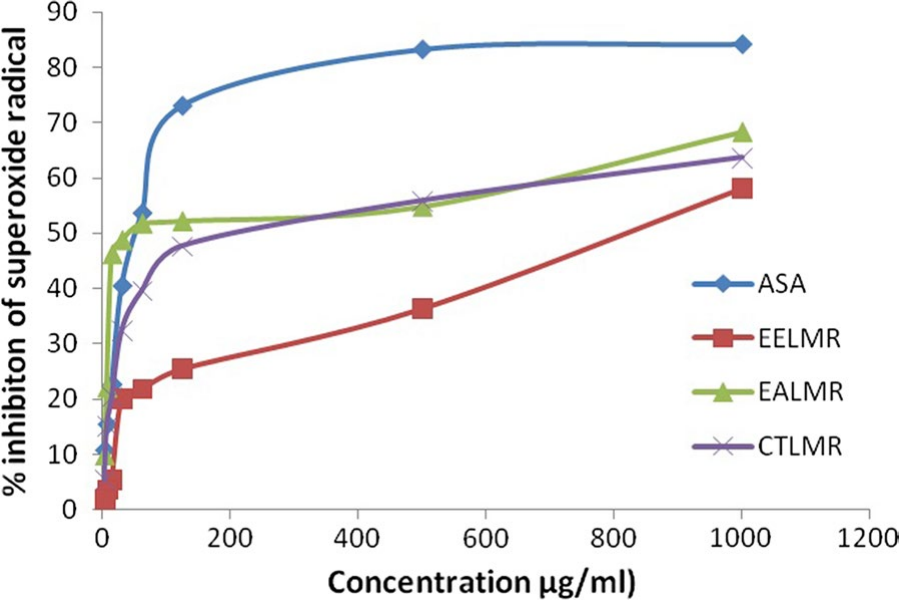
Superoxide radical scavenging activity of different extracts of roots of *L. macrophylla.* EELMR: The values are the average of duplicate experiments. Ethanolic extract of *L. macrophylla* roots; *CTLMR* carbon tetrachloride soluble partitionate, *EALMR* ethyl acetate soluble partitionate, *ASA* ascorbic acid

All the tested samples except ethanolic extract of roots of *L. macrophylla* significantly eliminated superoxide radical as shown in Table 5 and Fig. 3.

The ethyl acetate partitionate of the *L. macrophylla* showed the most potent scavenging activity with IC_50_ value 155.62 μg/ml which is comparable to that of ascorbic acid (IC_50_ value 99.66 μg/ml. Superoxide radical is involved in many pathological conditions. It mediates inflammatory tissue injuries in ischaemia–reperfusion, arthritis, gout, gastric ulceration. Superoxide radical has a low reactivity and a low capacity to penetrate the lipid membrane layer, but it can generate hydrogen peroxide and highly reactive hydroxyl radical, via Haber–Weiss reaction. Superoxide radical scavenging activity of these plant extracts can be explained by its polyphenolic content which scavenged directly superoxide radical by the hydrogen-donating capacity of their phenolic groups.

### Nitric oxide (NO) scavenging activity

**Fig. 4:**
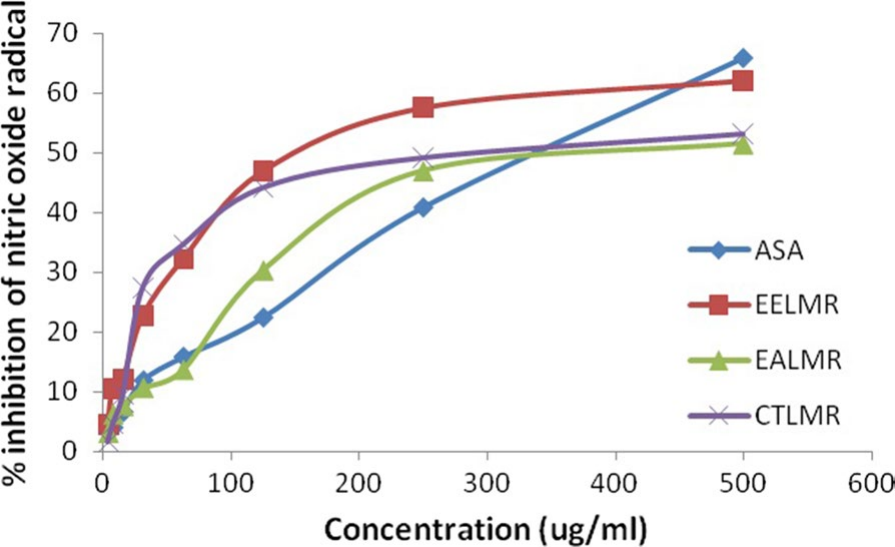
Nitric oxide (NO∙) scavenging activity of roots of *L. macrophylla*. The values are the average of duplicate experiments

Nitric oxide (NO) is a reactive free radical produced by phagocytes and endothelial cells to yield more reactive species such as peroxynitrite which can be decomposed to form OH∙ radical. The level of NO∙ was significantly reduced in this study by the ethanolic extracts of roots of *L. macrophylla* (with IC_50_ value of 210.12 μg/ml) and its ethyl acetate soluble partitionate (with IC_50_ value of 407.7 μg/ml) both of which are comparable to that of ascorbic acid (with IC_50_ value of 356.04 μg/ml) shown in Table 5 and represent in Fig. 4.

NO scavenging capacity of the extract may help to arrest the chain of reactions initiated by excess generation of NO that lead to various pathogenic pathways underlying a large group of disorders including muscle diseases, inflammatory bowel disease, sepsis and septic shock, primary headaches and stroke [21]. Additionally, increasing evidence shows that NO modulates neurotoxin-induced cell damage and is involved in neuronal cell death in Parkinson’s disease and other neurodegenerative disorders such as Alzheimer disease [20]. Potential NO scavenging activity of the plant may explicate the use of it for the treatment of inflammatory and neurological disorders.

### Determination of reducing power by potassium ferricyanide and trichloroacetate

**Fig. 5:**
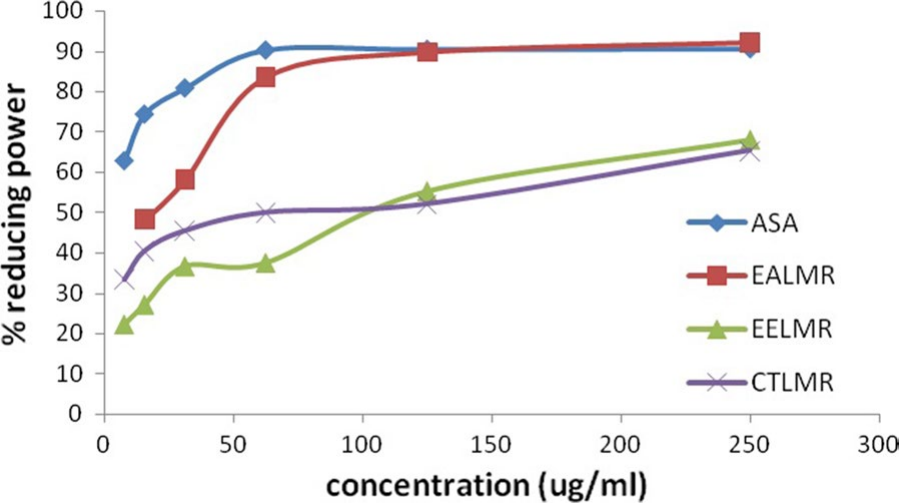
Reducing power assays of the different extracts of *roots of L. macrophylla* and the reference standards. The values are the average of duplicate experiments

In the reducing power assay, the presence of antioxidants (such as phenolic compound) in the sample would result in the reduction of Fe^3+^ to Fe^2+^ by donating an electron. The amount of Fe^2+^ complex can then be monitored by measuring the formation of Perl’s blue at 700 nm. Increasing absorbance indicates an increase in reductive activity. The results showed that there was increase in reducing power of all the plant extracts as the extract conc. increases in Fig. 5.

Among the tested extracts the ethyl acetate fraction of ethanolic extract of roots of *L. macrophylla* showed the highest reducing power (EC_50_ = 15.27 µg/ml) while the EC_50_ of the standard ascorbic acid was 0.91 µg/ml as Table 5.

This study showed that different extracts of roots of *L. macrophylla* possess potential DPPH, superoxide, and NO∙ free radical scavenging activities. The antioxidant activities of the plant extracts might be due to the presence of oleanolic acid, oleanolic acid derivative 7 α, 28-olean diol and stigmasterol. Previously it is reported that oleanolic acid plays as an antioxidant through quenching reactive oxygen species (ROS), inhibiting lipid peroxidation or stimulating cellular antioxidant defences [22, 23]. On the other hand, the phytosterol such as stigmasterol chemically acts as an antioxidant, a modest radical scavenger, and physically as a stabilizer in the membranes [24]. In conclusion, this study demonstrated that roots of *L. macrophylla* can be a very potential source of antioxidant agents.

## Conclusion

We have described the antioxidant activity of the roots of *L. macrophylla* (Roxb.) which is beneficial in the modern traditional medicine, validated in this study by in vitro DPPH, superoxide, and NO free radical scavenging activities of different organic fractions. However, roots of *L. macrophylla* showed the promising antioxidant effects to be studied further for therapeutic applications. The antioxidant activities of the plant extracts might be due to the presence of oleanolic acid, oleanolic acid derivative 7 α, 28-olean diol and stigmasterol. Identification of bioactive components claimed to use these indigenous sources for pharmaceutical preparations. Which may lead an advanced step for prognostic treatment of several diseases such as cancer, diabetes, aging and neurodegenerative disorders is the antioxidant mechanism that is developed for the protection of living systems against oxidative injuries caused by reactive oxygen and nitrogen species.
